# Interface induced diffusion

**DOI:** 10.1038/s41598-021-88808-1

**Published:** 2021-04-29

**Authors:** S. Gurbán, A. Sulyok, Miklos Menyhárd, E. Baradács, B. Parditka, C. Cserháti, G. A. Langer, Z. Erdélyi

**Affiliations:** 1 Thin Film Department, Centre for Energy Research, Institute for Technical Physics and Materials Science, P.O.B. 49, Budapest, 1525 Hungary; 2 Department of Solid State Physics, Faculty of Sciences and Technology, University of Debrecen, P.O. Box 400, Debrecen, 4002 Hungary; 3 Department of Environmental Physics, University of Debrecen, Poroszlay u. 6, Debrecen, 4026 Hungary

**Keywords:** Condensed-matter physics, Nanoscale materials

## Abstract

Interface induced diffusion had been identified in a thin film system damaged by electron bombardment. This new phenomenon was observed in Al_2_O_3_ (some nm thick)/Si substrate system, which was subjected to low energy (5 keV) electron bombardment producing defects in the Al_2_O_3_ layer. The defects produced partially relaxed. The rate of relaxation is, however, was different in the vicinity of the interface and in the "bulk" parts of the Al_2_O_3_ layer. This difference creates an oxygen concentration gradient and consequently oxygen diffusion, resulting in an altered layer which grows from the Al_2_O_3_/Si substrate interface. The relative rate of the diffusion and relaxation is strongly temperature dependent, resulting in various altered layer compositions, SiO_2_ (at room temperature), Al_2_O_3_ + AlO_x_ + Si (at 500 °C), Al_2_O_3_ + Si (at 700 °C), as the temperature during irradiation varies. Utilizing this finding it is possible to produce area selective interface patterning.

## Introduction

Diffusion decreases the difference in chemical potential, which, in the simplest cases, manifests as concentration gradient. Concentration gradient is naturally present if two (or more) materials of various composition become connected, or if foreign atoms are placed into a matrix (as in case of ion implantation amongst others).

It has long been established that electron irradiation might cause defects in solids initiating various alterations. Hong et al. applied electron beam irradiation to affect the grain growth of Ag layer to enhance the optoelectronic properties of the Ag reflector in light emitting diode^1^, Liu et al. observed electron radiation-induced material diffusion in nanostructured amorphous CoFeB thin film^2^, Messina et al. calculated the enhancement of diffusion due to the radiation induced point defects^3^, etc. In these cases, the diffusion was studied within the irradiated region. A different situation occurs if irradiation affects only a limited region of the material and the presence of the altered, damaged region initiates “normal” diffusion to or form the remaining, undisturbed part of the specimen. For example, Chih-Hao et al. showed^4^ that the interface exhibiting damage induced point defect gradient and/or strain might initiate even uphill diffusion.

In this paper we will describe a completely new process. It is also an interface induced diffusion process but instead of defect and/or strain accumulation, the interface promotes the diffusion because of the difference in the relaxation rates of the electron bombardment produced defects at the interface and in the bulk. This new process will be illustrated by a study conducted on an Al_2_O_3_/Si substrate sample. Aluminium oxide/silicon system is an important one since Al_2_O_3_ (a) is a high dielectric constant material which might replace SiO_2_^5^ (b) is used for passivation in photovoltaic applications^6,7^. Since this system might also experience electron bombardment its degradation is to be checked.

In our previous paper^8^ we have studied effect of electron bombardment on the Al_2_O_3_/Si substrate system at room temperature; in this paper we study the same system at various elevated temperature. A great variety of alterations occur when varying the sample temperature during irradiation, ranging from serious degradation of the sample (at 500 °C) to slight metallic Si diffusion to the nearly perfect Al_2_O_3_ layer (at 700 °C). To explain the experimental findings, we will focus on the interface induced diffusion process. Electron bombardment produces defects by braking chemical bonds which are partly healed by relaxation. However, in our case, the difference in the relaxation rates close to the interface and in the bulk resulted in an oxygen gradient in the layer. If the temperature of the sample is sufficiently high, oxygen diffusion occurs from the interface region to the free surface causing interface migration resulting in the growth of an altered layer. This newly discovered process offers for novel application as well. It allows a unique possibility to write at the Al_2_O_3_/Si interface with different “colors” creating double pattering via targeting selected regions using different temperatures during irradiation. Similar processes and wealth of possible applications are expected in nano materials having many interfaces.

## Results

In this study similar samples have been used as in the previous study^8^, where the initial state of the samples had been carefully characterized and published. In short: the in-depth composition has been determined by AES depth profiling (see depth profiles in Figs. 4a and 5a in ref 8) and the initial interface was found to be sharp with thickness of less than 0.5 nm. The interface consists a slight Si oxide contamination in the range of 0.1–0.3 monolayer. In case of all the reported experiments, the non-irradiated regions of the sample were also depth profiled and no deviations from the as received sample had been found.

### Irradiation at room temperature

**Figure 1:**
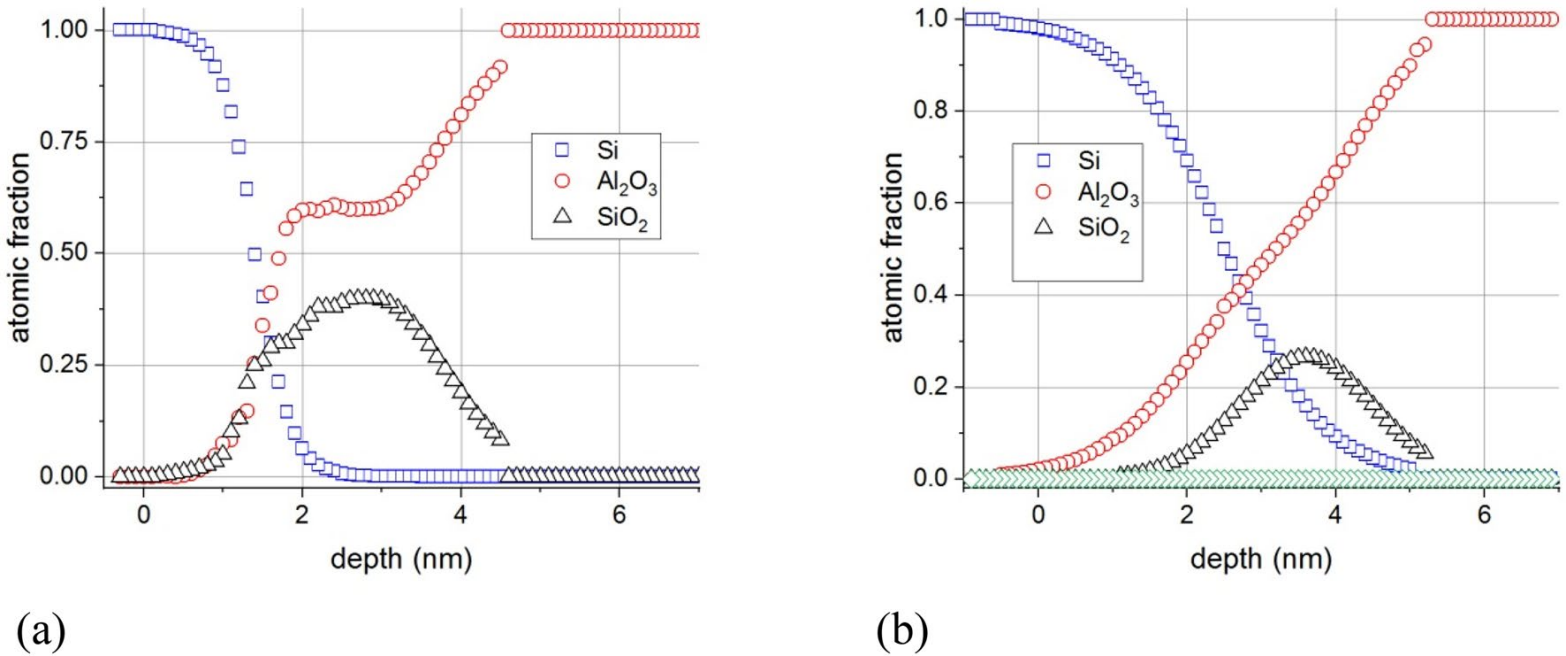
Concentration distributions after electron irradiation at room temperature of 5 keV, I = 500 nA, 21 h for samples (**a**) 5 nm Al_2_O_3_/Si and (**b**) 20 nm Al_2_O_3_/Si (the non-altered part of the sample is not shown). The position of the original Al_2_O_3_/Si interface is at 0 nm, the positive direction is toward the layer. The denotations Si, Al_2_O_3_, and SiO_2_ in the legend stand for pure Al_2_O_3_, (metallic) Si, and SiO_2_, respectively.

The effect of irradiation of the 5 nm Al_2_O_3_/Si substrate sample have been described in detail^8^. Since the effects of irradiation at elevated temperature were expected to be much stronger than that at room temperature, thus we also used thicker, 20 nm Al_2_O_3_/Si substrate sample. To check if the layer thickness affects the phenomena first we irradiated the 20 nm thick sample at room temperature. Figure 1a shows the in-depth concentration distributions determined on the sample of 5 nm Al_2_O_3_/Si substrate after 5 keV electron irradiation at room temperature, (I = 500 nA, 21 h), while Fig. 1b shows the in-depth concentration distributions obtained after similar electron irradiation on the sample 20 nm Al_2_O_3_/Si substrate. The origin of the depth scale is set to the adlayer/substrate interface; the positive and negative direction is toward the adlayer and substrate, respectively.

It can be seen that in the case of the 20 nm Al_2_O_3_/Si substrate sample the majority of the Al_2_O_3_ layer remained unchanged while close to the Si/Al_2_O_3_ interface a SiO_2_ layer forms due to electron irradiation. The formation of the SiO_2_ layer in the case of the other sample, 5 nm Al_2_O_3_/Si substrate (Fig. 1a), is qualitatively similar. Obviously here the majority of the Al_2_O_3_ layer is affected.

### Irradiation at elevated temperature

In these experiments the irradiated region was negligibly small compared to the size of the samples– it is in the range of 80–150 μm in diameter versus 1 cm^2^ the whole area—thus the neighbouring heated but not irradiated regions could be and were always simultaneously depth profiled to determine whether heating in itself causes detectable changes. We found that for any time duration and temperature combination of the heating (in the range of 20–750 °C), the non-irradiated regions of the sample (thus the major part) did not show any changes; the in-depth distributions recorded agreed well with that obtained on the pristine sample.

#### Irradiations at 500 °C

**Figure 2:**
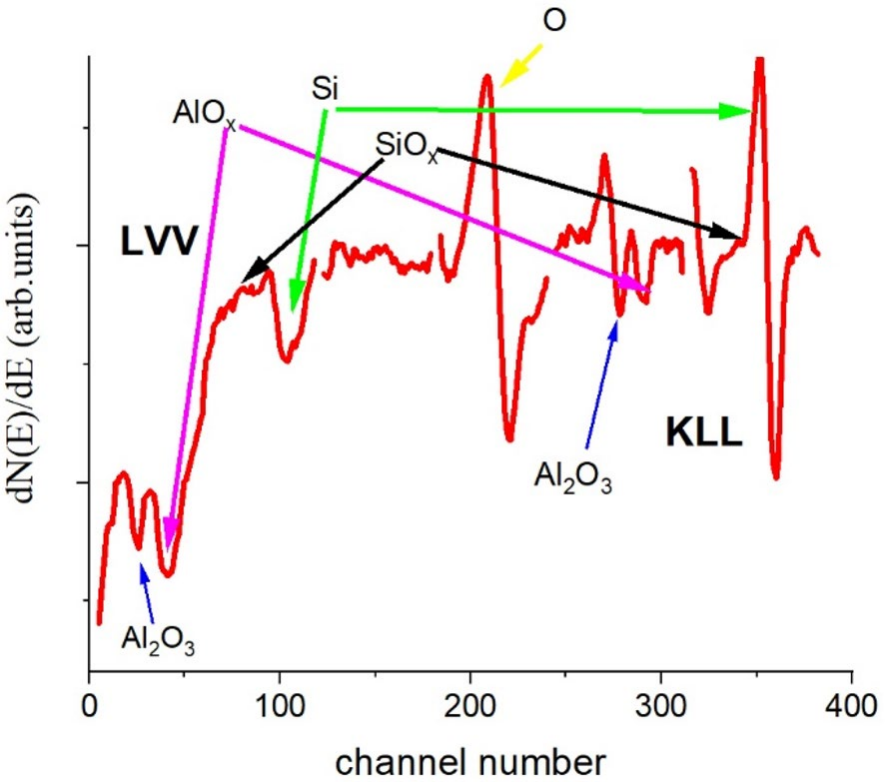
The differentiated, N(E)’, Auger electron spectrum of 5 nm Al_2_O_3_/Si substrate sample recorded in a depth of 3.2 nm (measured from the free surface) after the irradiation of 500 nA and 16 h. The temperature of the sample during irradiation was 500 °C. AlO_x_ stands for Al suboxide. The black arrows labelled SiO_x_ show the position where the silicon suboxide peak should be (in fact here they are absent). The peaks belonging to LVV and KLL transitions are indicated.

If irradiation took place at 500 °C for sufficiently long time, the Al_2_O_3_/Si substrate system exhibited serious changes. To demonstrate this, the as-recorded differentiated Auger spectrum is shown in Fig. 2 which was obtained in a depth of 3.2 nm (measured from the free surface) on sample of 5 nm Al_2_O_3_/Si substrate after 16 h irradiation with 500 nA.

**Figure 3:**
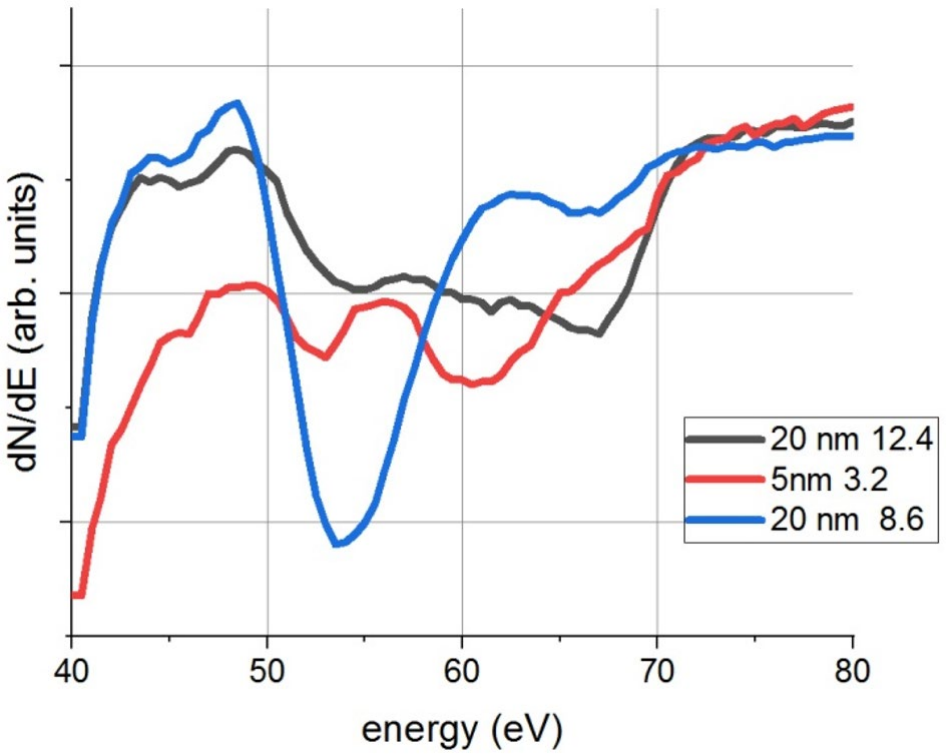
Al_LLV_ lines in various depths (measured from the free surface) and samples (with 20 nm and 5 nm thick Al_2_O_3_ layers, resp.); the last number in the legend gives the depth (in nm, measured from the free surface) of the region providing the Auger line.

According to Fig. 2 the originally pure Al_2_O_3_ layer drastically changed due to irradiation. Metallic Si (not in oxide state) develops in the originally pure Al_2_O_3_ matrix and besides the characteristic Al-oxide Auger peaks (LVV and KLL), new features appear in the spectrum, indicating the presence of sub-stoichiometric aluminium oxide (suboxide) – which will be denoted as AlO_x_^−^, where 0 < x < 2, There is no silicon oxide and/or suboxide (denoted from here on as SiO_x_).The shapes and positions of the KLL and LVV transitions of both Al and Si strongly affected by the oxidation state. It should also be emphasized that for suboxides the above energies are different; the change is larger for the LVV (containing valence transitions) Auger peaks than that of the KLL Auger peaks. Thus, the Al_LVV_ line will be used to follow the chemical change along the depth. Some examples (taken from depth profile of samples with 20 and 5 nm thick Al_2_O_3_, resp.) are shown in Fig. 3.

It is clear that the chemical state of the Al varies with depth. In the following figures only two Al related concentrations will appear; one for the stoichiometric Al_2_O_3_ state and one for the remaining aluminium oxides, that is, for all different kinds of AlO_x_ suboxides (containing various oxygen deficits).

**Figure 4:**
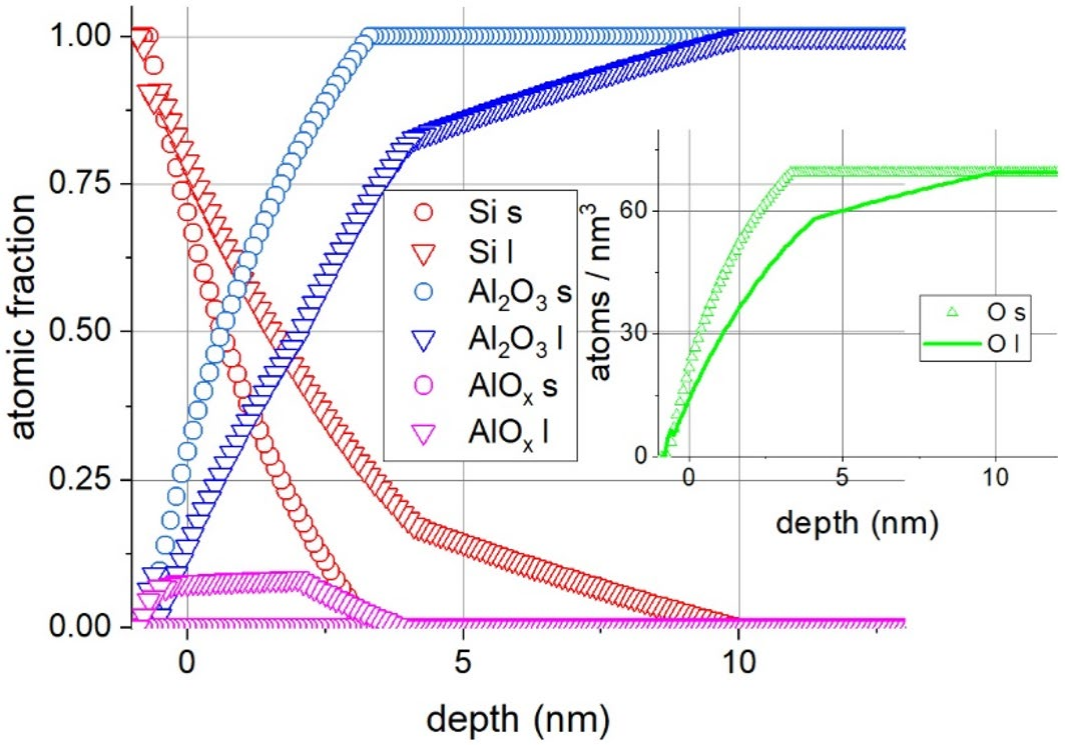
Depth profiles of 20 nm Al_2_O_3_/Si substrate samples irradiated by Q (denoted by s in the legend) and 2Q (denoted by l in the legend) electron dose; sample temperature is 500 °C. The position of the original Al_2_O_3_/Si interface is at 0 nm, the positive direction is toward the layer (for clarity only the altered part is shown). The insert shows the O depth profile in atoms/nm^3^ units.

The amount of damage created by the electron bombardment also depends on the dose of the irradiation. This is demonstrated in Fig. 4. that shows the concentration distributions vs. depth for irradiation by Q and 2Q electrons denoted by *s* and *l*, respectively in the case of a 20 nm Al_2_O_3_/Si substrate sample.

**Figure 5:**
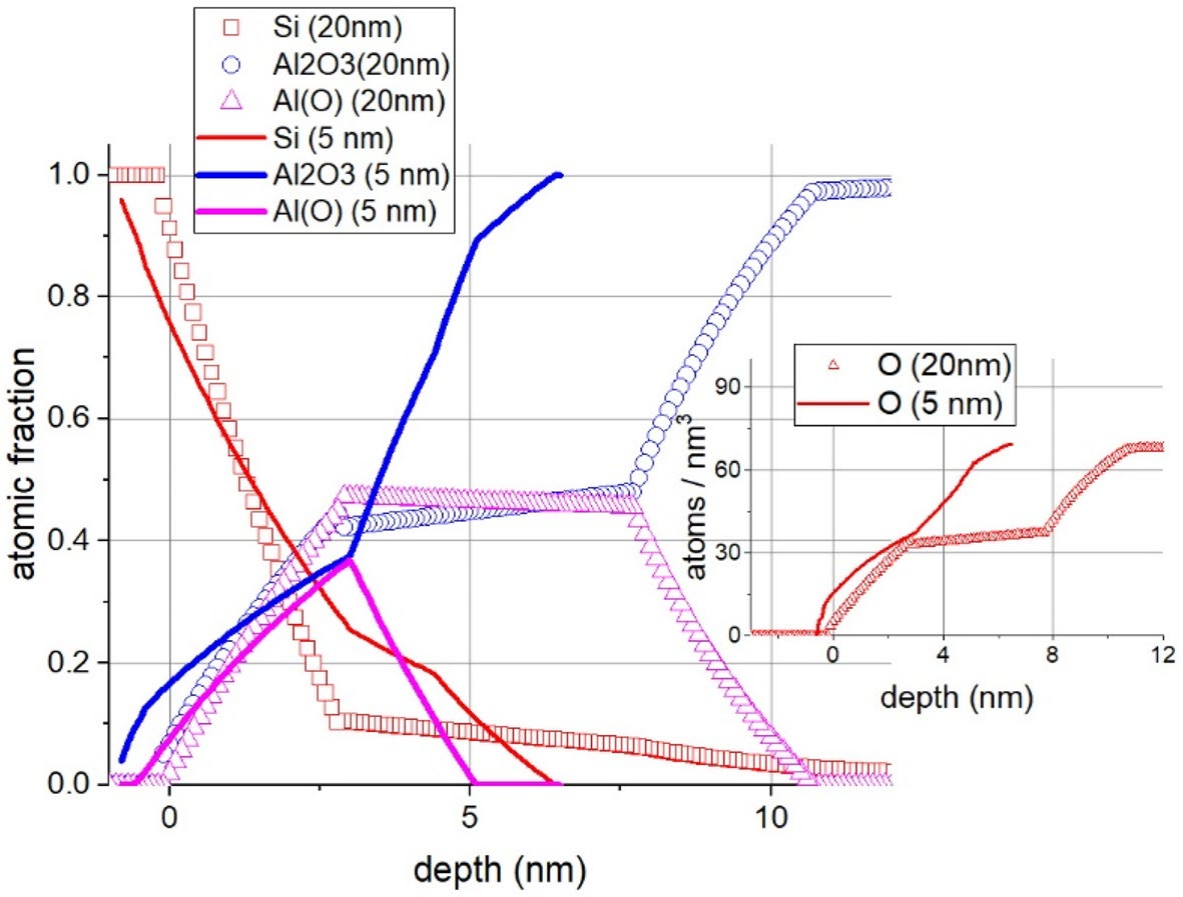
The concentration distributions after electron irradiation at 500 °C (5 keV, I = 500 nA, 20 h ) of samples 5 nm and 20 nm Al_2_O_3_/Si substrate. The position of the original Al_2_O_3_/Si interface is at 0 nm, the positive direction is toward the layer (for clarity only the altered part is shown). The denotations Al_2_O_3_, Si, AlO_x_ in the legend stand for pure Al_2_O_3_, metallic Si and Al suboxide, respectively. The insert shows the O depth profile in atoms/nm^3^ units.

Figure 4 demonstrates that irradiation with higher dose causes more extensive alterations. The effect of irradiation also depends on the thickness of the Al_2_O_3_ layer (see Fig. 5).

Qualitatively, the two samples show similar results, which can be summarized as follows:

- a layer of the mixture of AlO_x_ and metallic Si grows from the Al_2_O_3_ /Si interface toward the free surface.
- there is SiO_2_ neither in the layer nor at the Al_2_O_3_ /Si interface
- the oxygen (in oxide bond) decreases toward the interface

#### Irradiations at 700 °C

**Figure 6:**
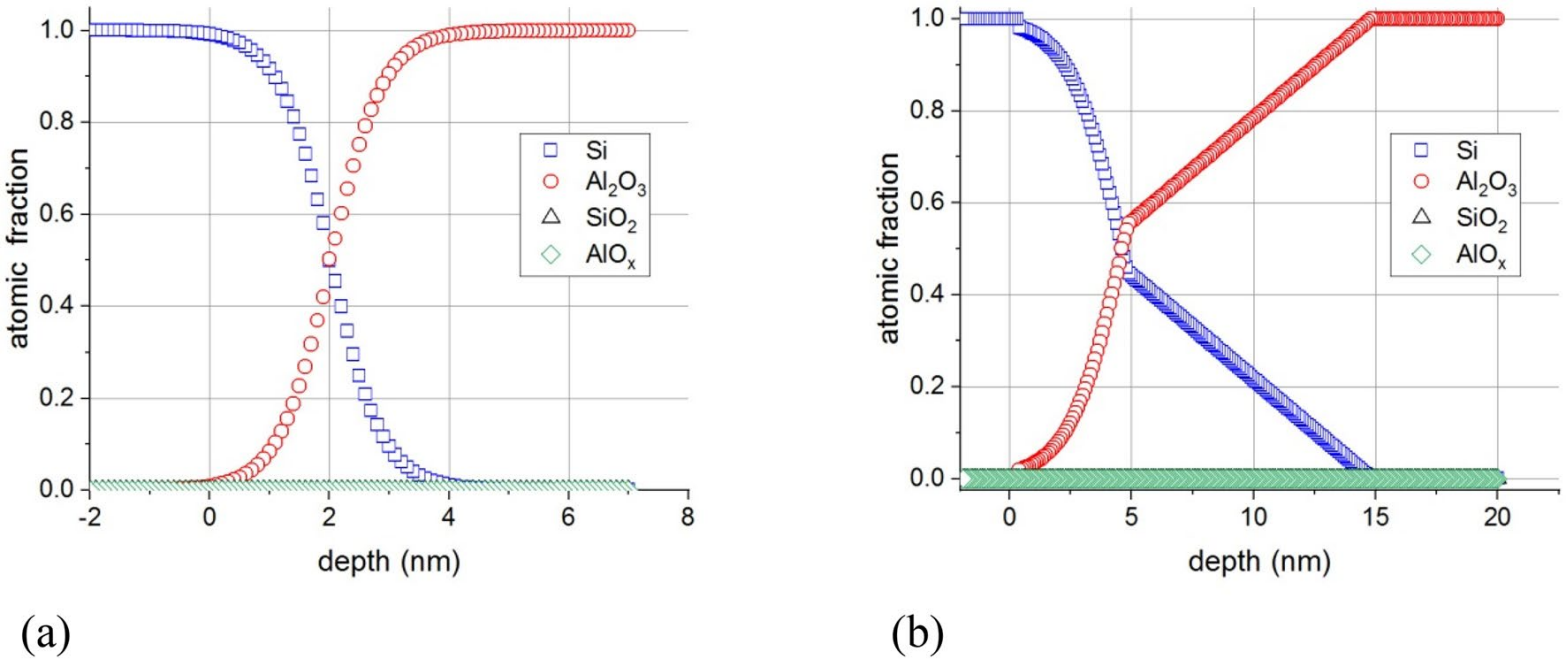
Concentration distributions after electron irradiation at 700 °C of sample (**a**) 5 nm Al_2_O_3_/Si substrate and (**b**) 20 nm Al_2_O_3_/Si substrate. The position of the original Al_2_O_3_/Si interface is at 0 nm, the positive direction is toward the layer.

At 700 °C, irradiation leads to another type of alterations, as it is shown in Fig. 6.

In contrast to the alteration which is produced by the irradiation if the sample temperature is 500 °C, in this case the Al_2_O_3_ layer seems to be untouched, consequently there is no oxygen diffusion. On the other hand, metallic silicon diffuses into the Al_2_O_3_ layer. Note that the sensitivity of the AES is around 5–7 at.%.

## Discussion

Let us first summarize the experimental findings.

First let us emphasize that the non-irradiated area for any heating temperature and time combination remained unchanged; the measured depth profiles were identical to those recorded on the as received samples.

On the other hand, the surface region of the sample irradiated by electrons showed various alterations. Generally: (a) in all experiments there is always a thin region connected to the free surface which is free from any alteration, (b) all alterations scale with the dose of electron irradiation.

The alteration is strongly temperature dependent:

- at room temperature SiO_2_ is produced at the interface^8^,
- at 500 °C AlO_x_ suboxide (0 < x < 2) and metallic Si mixture appears in the Al_2_O_3_ layer growing from the Al_2_O_3_ /Si interface, accordingly, the bound oxygen concentration decreases toward the Al_2_O_3_ /Si interface,
- at 700 °C the Al_2_O_3_ layer seems to be untouched, but Si diffuses into the Al_2_O_3_ layer.

We will show in the following that all these experimental findings, which seem to be very complex, can be explained by a simple model. The model considers only three elementary processes as (a) primary defect production, (b) primary relaxation, (c) diffusion.

Before dealing with the alterations obtained, the basic feature of the Al_2_O_3_ layer is to be considered. Al_2_O_3_ layers are frequently used as a protection on Si based photovoltaic devices^6,7^; accordingly, the expectation is that they do not interact with the underlying Si. This was what we found in case of our samples, too.

### The effect of electron irradiation; primary defect production

The excitation of defects has been discussed in our previous paper^8^. Its essence is the following: from the various possible interactions (heating, knock-on ionization, electronic excitation etc.) in the present case the radiolytic processes are active^9^, similar to that seen in low-energy electron-stimulated desorption (ESD)^10,11^ experiments. Thus, electron irradiation produces neutral and/or charged O in the Al_2_O_3_ matrix together with various charged and neutral crystal defects.

The excitation is temperature independent and are produced by the primary electron beam current and the backscattered secondary electrons; the intensities of which do not vary along the 20 nm or 5 nm thick Al_2_O_3_ layer. Thus, in the investigated systems, the excitation is temperature and depth independent; the primary excited defects and excited O species are homogeneously distributed in the Al_2_O_3_ layer.

### Primary relaxation process

The change in the number of defects in a volume element *ΔV* in any moment is given by the difference between the production and annihilation of the defects. The number of defects produced by electron bombardment in *ΔV* volume of the "bulk" part of the Al_2_O_3_ matrix during *Δt* time is ${I}_{e}\Delta tAd{\sigma }_{e}$*,* where *I*_*e*_ is the bombarding electron current, *A* is the cross section of the electron beam, *d* is the thickness of the layer, while *σ*_*e*_ is the ionization cross section. Obviously some of the defects can recover; it is supposed that the relaxation of defects depends on the number of defects. Thus, the change of the number of defects can be given as:

1a $$ \frac{d\left({\rho }_{Al}\Delta V\right)}{dt}={s}_{d}-\Delta V{\rho }_{Al} \, {\text{exp}}\left(-\frac{Q_{\text{Al}}}{kT}\right)$$

where ${\rho }_{Al}$ is the defect concentration (volume density) in the Al_2_O_3_ matrix and *Q*_*Al*_ is the activation energy for aluminum-oxide formation and ${s}_{d}$ is the rate of defect production by irradiation. The solution of this equation is

1b $${\rho }_{Al}\Delta V=\frac{{s}_{d}}{{\lambda }_{Al}}\left[1-\mathrm{exp}\left(-{\lambda }_{Al}t\right)\right]$$

with ${\lambda }_{Al}=\mathrm{exp}\left(-\frac{{Q}_{Al}}{kT}\right)$. Thus, the defect concentration increases until the rate of defect relaxation becomes equal to the rate of defect production, at which point a dynamic equilibrium is reached ($t\to \infty$ in Eq. (1b))

1c $${\rho }_{Al}^{stat}=\frac{{s}_{d}}{{\lambda }_{Al}}$$

Obviously, the stationary concentration of defects is temperature dependent.

In the surrounding of the adlayer/substrate interface the above process is disturbed by the appearance of a new agent, the Si-which can also bind the excited oxygen. Since a new recombination center appears, we should modify Eq. (1a) as

2a $$\frac{d\left(\rho\Delta V\right)}{dt}={s}_{d}-\Delta V\rho \left[{c}_{Al}\mathrm{exp}\left(-\frac{{Q}_{Al}}{kT}\right)+{c}_{Si}\mathrm{exp}\left(-\frac{{Q}_{Si}}{kT}\right)\right]$$

where *Q*_*Si*_ is the activation energy for silicon oxide formation, furthermore ${c}_{Al}$ and ${c}_{Si}$ are the fraction of Al_2_O_3_ and Si in $\Delta V$ volume. This activation energy is higher (see later) than that of *Q*_*Al*_, accordingly, the rate of the defect relaxation decreases and thus the stationary defect (excited oxygen) concentration is higher than that in the "bulk" part of the layer. The solution of Eq. (2a) is.

2b $$\rho\Delta V=\frac{{s}_{d}}{{{c}_{Al}\lambda }_{Al}+{{c}_{Si}\lambda }_{Si}}\left\{1-\mathrm{exp}[-\left({{c}_{Al}\lambda }_{Al}+{{c}_{Si}\lambda }_{Si}\right)t]\right\}$$

where ${\lambda }_{Si}=\mathrm{exp}\left(-\frac{{Q}_{Si}}{kT}\right)$, with stationary defect concentration of

2c $${\rho }^{stat}=\frac{{s}_{d}}{{{c}_{Al}\lambda }_{Al}+{{c}_{Si}\lambda }_{Si}}$$

Note that ${\rho }_{Al}^{stat}<{\rho }^{stat}$ if ${Q}_{Al}<{Q}_{Si}$.

### Diffusion

Based on the above—because of the presence of the interface—a concentration gradient of excited oxygen is built up. The diffusivity (*D*) of oxygen in Al_2_O_3_ is in the range of 4.6 × 10^−34^–1.3 × 10^−32^ m^2^/s, and 2.9 × 10^−22^–1.1 × 10^−21^ m^2^/s, at room temperature and at 500 °C, respectively^12,13^.

Now we will explain the experimental findings based on the above.

#### General observations (experimental findings a and b)

- In all of our experiments, we found an unchanged region connected to the free surface. Thus, in this region the concentration of defects (if they exist at all) is less than a few percentage, which is the sensitivity level of our analysis. To explain this finding we must suppose that *Q*_*Al*_ in the surface close region is lower than that in the "bulk" since in this region atomic reconstruction is possible.
- The number of defects is proportional to the exciting current [Eqs. (1) and (2)] and, as all processes depend on the number of defects, dependence on the dose is evident.

#### Alteration as a function of temperature

##### Room temperature

At room temperature irradiation SiO_2_ formation occurs. At room temperature there is excited oxygen at the interface, which cannot depart because of its low diffusion rate (at room temperature the diffusion length $\left(\sqrt{Dt}\right)$ of excited oxygen is in the range of 6.3 × 10^−6^–3.3 × 10^−5^ nm/day^12,13^, and thus it interacts with the Si and oxide formation occurs. This oxide can have a limited growth according to the Cabrera and Mott process^14^ detailed in our previous paper^8^. The amount of oxide produced does not depend on the actual layer thickness since only the primary excitation is to be considered, which is homogeneous along the depth.

##### 500 °C

In contrary to the modest changes at room temperature, at 500 °C net oxygen loss and consequently a mixture of Al_2_O_3_, Al suboxide (AlO_x_) layer formation, and Si diffusion toward the free surface occur. Here we should consider that at 500 °C the excited oxygen becomes mobile; its diffusion length is in the range of 5–10 nm/day allowing oxygen transport.

In the vicinity of the interface the concentration of excited oxygen is higher than that in the "bulk" Al_2_O_3_ because of the higher activation energy of the silicon oxide formation, thus an oxygen gradient toward the free surface builds up. At this temperature the "physisorbed" oxygen easily desorbs to the vacuum from the surface of the Al_2_O_3_, thus electron irradiation induced oxygen current develops and a net oxygen loss appears. As the oxygen diffuses away, a region with sub-stoichiometric AlO_x_ appears with varying oxygen content (see Fig. 2 and 3.) This region, attached to the Si substrate, contains various defects and induces enhanced metallic Si diffusion. This observation corroborates the assumption that the activation energy of the Si–O bond formation is higher than that of Al-O; in this defected region in the presence of AlO_x_ the metallic Si has no chance for making Si–O bond. The defected region grows into the originally pure Al_2_O_3_ matrix (see Fig. 4) and thus the interface also migrates. Thus, the region with higher excited oxygen concentration than that of pure Al_2_O_3_ also migrates and drives the diffusion. This oxygen "pump" is so effective that even the oxygen, which was initially present on the Al_2_O_3_/Si substrate interface is also carried away.

##### 700 °C

If electron irradiation occurs at 700 °C the Al_2_O_3_ seems to be unhurt, that is the number of defects is lower than 5% (the sensitivity of the AES is around 5%) and Si diffusion occurs. Evidently, primary defect formation is active, thus excited oxygen is produced by the irradiation. Its stationary level is, however, strongly temperature dependent (see Eqs. (1c) and (2c)). Taking, for example, Eq. (1c), we can estimate the factor by which the stationary level of defect density is decreased due to the increase of the temperature:

3 $$\frac{{\rho _{{Al}}^{{stat}} \left( {T_{h} } \right)}}{{\rho _{{Al}}^{{stat}} \left( {T_{l} } \right)}} = \exp \left[ {\frac{{Q_{{Al}} }}{R}\left( {\frac{1}{{T_{l} }} - \frac{1}{{T_{h} }}} \right)} \right].$$

Considering that the activation energy is in the order of eV and that *T*_*h*_ = *700* °C and *T*_*l*_ = *500* °C, we obtain that the decrease is at least one to two orders of magnitude. Similar factor is obtained for the interface region, that is for $\frac{{\rho }^{stat}\left({T}_{h}\right)}{{\rho }^{stat}\left({T}_{l}\right)}$. Accordingly, the concentration gradient of excited oxygen is also decreased by at least one to two orders of magnitude, and thus the driving force for diffusion practically vanishes. This is in agreement with the experimental finding that no defects (higher than 5%) were observed. It should be concluded that the relaxation process is faster than that of diffusion, thus practically all oxygen binds back before the diffusion can transport it away.

Still thermally activated Si diffusion occurs. Here we note that Si movement was also observed at room temperature since SiO_2_ formation occurred, that movement was, however, not thermally activated diffusion but following the Cabrera-Mott process (see also ref. 8). To explain the observed diffusion, we recall that defect production is temperature independent thus defect formation occurs during irradiation at 700 °C. According to Eq. (1) to reach the stationary state the presence of some defects is necessary, thus the Al_2_O_3_ layer should contain defects. We have not found diffusion data for the Si/Al_2_O_3_ system but it is safe to suppose that at this higher temperature defect enhanced Si diffusion occurs.

By fitting the Si distribution profiles in Fig. 6, assuming that they were produced by diffusion, we may deduce the interdiffusion coefficient for the defected Al_2_O_3_/Si.

**Figure 7:**
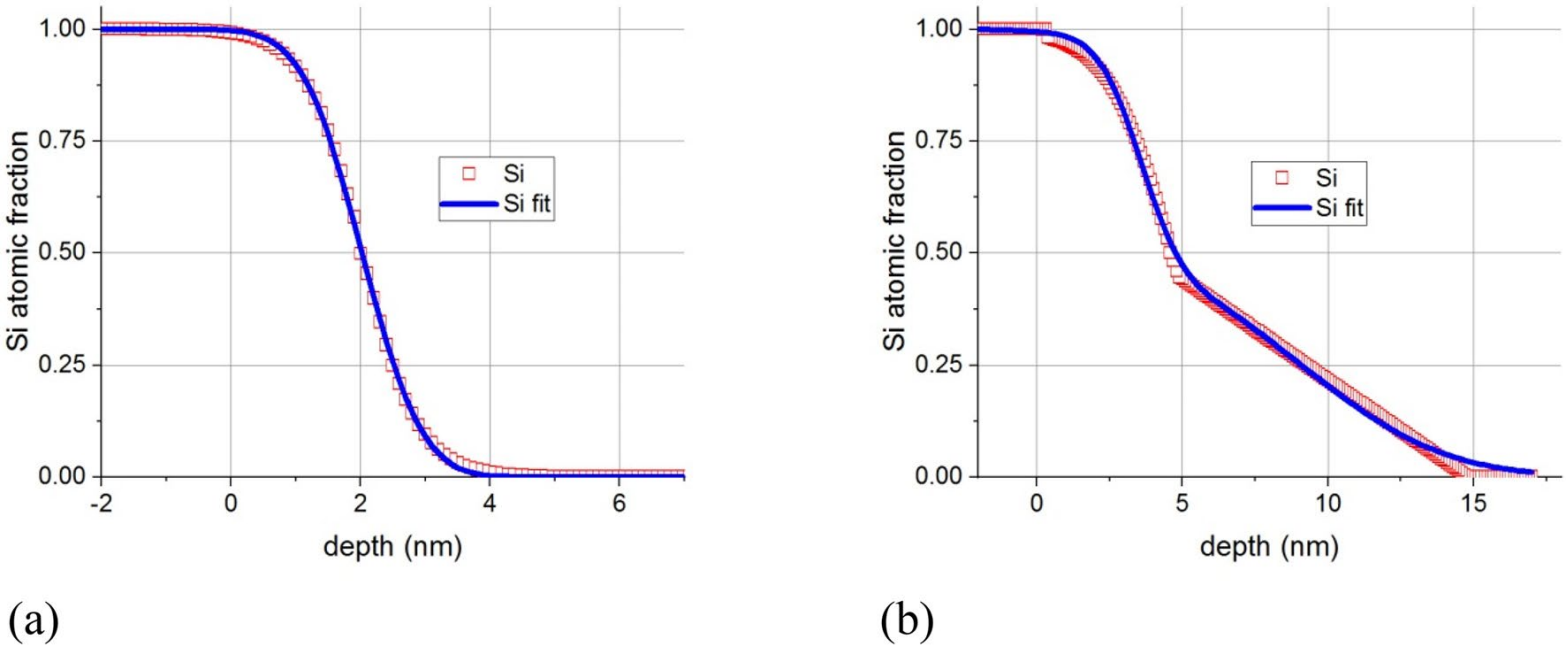
Fit of the composition profiles by erfc function to deduce the diffusion coefficients, for samples irradiated at 700 °C: (**a**) 5 nm Al_2_O_3_/Si substrate, (**b**) 20 nm Al_2_O_3_/Si substrate. Solid lines are the fitted curves.

Time development of the composition profile in Fig. 6a can be obtained from the solution of Fick’s second equation when the initial condition is a step function^15,16^.

4 $$c\left(x,t\right)=\frac{1}{2}\mathrm{erfc}\left(\frac{{x-x}_{0}}{2\sqrt{Dt}}\right),$$

where $c$ is the atomic fraction of the diffusion specie and ${x}_{0}$ is the position of the center of the profile (Matano plane). Fitting this to the composition profile of Si in Fig. 6a, a value of $2.7\times {10}^{-24}$ m^2^/s is obtained for diffusion coefficient. (see Fig. 7a).

We can see, however, that the composition profile in Fig. 6b cannot be fitted by a single error function. This diffusion process seems to be governed by two activation energies; typical for short-circuit diffusion (e.g. grain boundaries). For this reason, as a simple model, instead of using a single erfc function, we fitted the depth profile by a function consisting of two erfc functions containing two diffusion coefficients; one for the bulk diffusion $(D)$ and one valid in the short circuit $({D}^{^{\prime}})$. Although this is an oversimplified model—important structural parameters to build a robust model are unknown, it gives surprisingly reasonable results. (see Fig. 7b) The bulk diffusion coefficient is $7.9\times {10}^{-24}$ m^2^/s, whereas the short circuit one is ${D}^{^{\prime}}=1.2\times {10}^{-22}$ m^2^/s. As can be seen, the values of the bulk diffusion coefficients are practically the same for the two samples. The short-circuit diffusion is explained by the imperfections built in the sample; e.g. pinholes are typical in Al_2_O_3_. In the 5 nm distance range from the interface, the volume and short-circuit diffusion overlap, while the Si can reach farther than 5 nm only by short-circuit diffusion.

To conclude the discussion, we note that according to Eqs. (1) and (2) the lower the temperature the higher the stationary concentration of excited oxygen is. This means that the highest level is expected at room temperature, hence the gradient of the defects is also the highest. At this temperature, however, the oxygen is immobile, consequently they cannot depart from the interface but can react with the silicon atoms being there, resulting in a SiO_2_ layer. At 500 °C the oxygen is already mobile, the saturation density of the excited oxygen is still high enough to be detected and also its gradient large enough to provide sufficient driving force for diffusion. As a consequence, oxygen diffusion starts toward the free surface. No reaction with Si is possible, even oxygen debt is observed in the interface region. At 700 °C the density of the exited oxygen is low, cannot be detected, which also means that its gradient is negligible, so although oxygen would be mobile, the driving force for diffusion is missing. As a result, the excited oxygen stays in place and binds back quickly. Si can, however, move already at this temperature in the defected Al_2_O_3_, although the level of the defects is low, but definitely higher than without irradiation.

### Area selective double interface patterning

Finally, we will propose an application of the observed phenomenon.

It was shown that due to the electron irradiation a well-defined layer grows at the interface, which is highly stable. The composition of the layer, however, depends on the temperature during irradiation; at room temperature, 500 °C and 700 °C, SiO_2_, Al with AlO_x_ and Si form, respectively. This gives us the unique possibility to make various templates of different “colors” on an interface. We may first write a pattern by the piloted e-beam at the interface at room temperature (first pattern in color 1) then at 500 °C (second pattern in color 2), and finally at 700 °C (third pattern in color 3).

## Conclusions

5 and 20 nm Al_2_O_3_/Si substrate layer systems were irradiated by 5 keV electrons up to a dose of 3 × 10^7^ e/nm^2^ at various temperatures in the range of 20–700 °C. The layer system was stable; the non-irradiated regions of the sample were not affected by the heating. On the other hand, electron irradiation affected the layer system. The irradiation induced various alterations depend on the irradiation dose and temperature but the modified zone was always nucleated at the interface. At room temperature, irradiation a thin (2–3 nm) SiO_2_ was produced independently from the thickness of the initial Al_2_O_3_ layer. If the irradiation took place at 500 °C, a mixture of Al_2_O_3_ and AlO_x_ (0 < x < 2) were produced and simultaneously Si diffusion toward the free surface occurred along a considerable loss of O. This damaged region grows (from the interface toward the free surface) with increasing irradiation dose. If the irradiation took place at 700 °C, the integrity of the Al_2_O_3_ layer was only slightly affected and defect enhanced Si diffusion toward the free surface was observed.

A simple model has been developed to describe the findings, considering electron bombardment induced bond breaking, relaxation and diffusion describes all the experimental findings. Its essence is that the relaxation of the defects in the interface region is different from that of the bulk resulting in an excess excited oxygen concentration in the interface region because of the presence of the interface, that is, all processes observed are prompted by the presence of the interface. This excess oxygen concentration drives the O diffusion toward the free surface. The outcome depends on the relative rate of diffusion transport and relaxation. At around 500 °C the rate of relaxation is lower than the rate of diffusion resulting in a net oxygen loss and formation of aluminium suboxide. At 700 °C the rate of relaxation is higher than the rate of diffusion resulting in low density of the exited oxygen, which also means that its gradient is negligible, so although oxygen would be mobile, the driving force for diffusion is missing. On the other hand, the presence of a low concentration defects results in defect enhanced Si diffusion.

It was shown, that the temperature dependent phase transformation at the interface gives us the unique possibility to write at the Si/Al_2_O_3_ interface with different “colors” resulting in a various pattering.

## Samples and methods

### Sample

Samples were made by growing an Al_2_O_3_ layer on a Si (100) substrate using atomic layer deposition (ALD); for the details see ref 8.

### Electron irradiation

All electron irradiation experiments have been carried out in our standard Auger Electron Spectroscope (AES), using a standard electron gun; its parameters are: energy 0.1–10 keV, beam current 0.1–500 nA, beam diameter (energy dependent) 10–100 μm, scanning area up to 3 × 3 mm^2^. Because of the low efficiency of the process long irradiation times (16–28 h) were applied. As the long-term geometrical stability of our system is not sufficiently good various irradiation protocols have been applied. Generally, the beam scanned a small area, this method proved to be rather reliable but obviously the total dose per unit area is not maximal. If we wanted to apply the highest possible dose, then the beam was standing in a spot. The beam can jump between two points; the irradiation times at the two points are different. Obviously in this case again the total dose is far less than maximal. On the other hand, this mode provided excellent results concerning the dose dependence of the process. Based on the previous it is clear that the error of the value of the total irradiated dose is high. Typical irradiation current density at standing in one spot (applying 500 nA current with 100 μm diameter) is about 400e/s/nm^2^. This number seems to be high but if we consider that the cross section for ionization is in the range of 10^−16^ cm^2^, while the time for primary relaxation (see later) is in the range of 10^−12^ s, we conclude that the interaction events are independent.

The samples were mounted on a sample holder the temperature of which could be varied in the range of 20–800 °C.

### AES analysis

Two types of Auger analysis were applied. During the irradiation at any temperature the surface concentration was monitored. This measurement provides interesting data in the case of the 5 nm thick sample since all KLL Auger electrons emitted any depth of the layer and the surface close region of the substrate partly leave the sample and can be analysed. This measurement provides a rough description of the of the time evaluation of the alterations. On the other hand, it cannot provide detailed information on the concentration distributions along the depth. The latter can be obtained by AES depth profiling. All depth profiles were recorded on room temperature samples, where the conditions are frozen in. Thus the recorded depth profiles provide the state of the concentration distributions after a given irradiation.

For AES analysis the same electron gun was used for the excitation with a primary current and energy of 50 nA and 5 keV, respectively. The low irradiation time and current do not cause additional alterations in the sample.

The Auger spectra, N(E), were recorded by a pre-retarded cylindrical mirror analyser (DESA 150, Staib) in counting mode. The recorded spectrum was numerically differentiated for calculating the concentration.

The following Auger signals were measured: Al_KLL_, Al_LVV_, Si_KLL_, Si_LVV_, all cases in metallic and oxide forms, C and O. The escape depths of the Auger electrons depend on their energy and the matrix they travel. Si and Al are neighbouring elements and the energy of their LVV and KLL Auger electrons are close. In oxide form there is a larger change (about 14 eV) of the energy of the Auger electron; thus it is easy their distinction in analysis but this change from the point of view of inelastic mean free paths (IMFP) is small. Similarly, the IMFPs are rather close in SiO_2_ and Al_2_O_3_; the difference is 20 and 10% for the LVV and KLL Auger electrons, respectively. Thus, for all LVV and KLL Auger electrons travelling in any available matrix the IMFPs are around 0.7 nm and 3.3 nm, respectively^17^. By measuring the intensity of the high energy (KLL) and low energy (LVV) Auger electrons simultaneously, a rich data state is obtained, which improves the accuracy of the determination depth distributions of the concentrations. The error of the concentration values is about 7%.

### AES depth profiling

The parameters of the ion bombardment used for AES depth profiling were: energy 1 keV, projectile Ar^+^, angle of incidence 80° (with respect to the surface normal) and specimen rotation during ion bombardment. The ion beam was scanned in an area of 1.5 × 1.5 mm^2^. Using these parameters, the ion bombardment induced roughening and mixing is minimal, and a resolution of less than 1 nm can be reached^18^ .

### Determination of the concentration distribution from AES spectra

This was made exactly the same way as in ref 8. Its summary is the following: Instead of the usual relative sensitivity factor based routine^19^ we applied our trial and error approach to determine the composition distribution of such samples^20^. The essence of this method is that we assume a composition distribution along the depth, with steps of 0.1 nm, and calculate the Auger intensities assuming that the transport of electrons can be described by the exponential attenuation law. The composition distributions are varied until the simulated depth profile is close enough to the measured one. If one detects high (high IMFP) and low energy (low IMFP) Auger electrons, as in the present case, the accuracy of the method is rather good.
